# SurfNetv2: An Improved Real-Time SurfNet and Its Applications to Defect Recognition of Calcium Silicate Boards

**DOI:** 10.3390/s20164356

**Published:** 2020-08-05

**Authors:** Chi-Yi Tsai, Hao-Wei Chen

**Affiliations:** Department of Electrical and Computer Engineering, TamKang University, New Taipei City 251, Taiwan; cecer0530@gmail.com

**Keywords:** deep learning, supervised end-to-end learning, surface defect recognition, SurfNet, calcium silicate boards

## Abstract

This paper presents an improved Convolutional Neural Network (CNN) architecture to recognize surface defects of the Calcium Silicate Board (CSB) using visual image information based on a deep learning approach. The proposed CNN architecture is inspired by the existing SurfNet architecture and is named SurfNetv2, which comprises a feature extraction module and a surface defect recognition module. The output of the system is the recognized defect category on the surface of the CSB. In the collection of the training dataset, we manually captured the defect images presented on the surface of the CSB samples. Then, we divided these defect images into four categories, which are crash, dirty, uneven, and normal. In the training stage, the proposed SurfNetv2 is trained through an end-to-end supervised learning method, so that the CNN model learns how to recognize surface defects of the CSB only through the RGB image information. Experimental results show that the proposed SurfNetv2 outperforms five state-of-the-art methods and achieves a high recognition accuracy of 99.90% and 99.75% in our private CSB dataset and the public Northeastern University (NEU) dataset, respectively. Moreover, the proposed SurfNetv2 model achieves a real-time computing speed of about 199.38 fps when processing images with a resolution of 128 × 128 pixels. Therefore, the proposed CNN model has great potential for real-time automatic surface defect recognition applications.

## 1. Introduction

**Figure 1:**
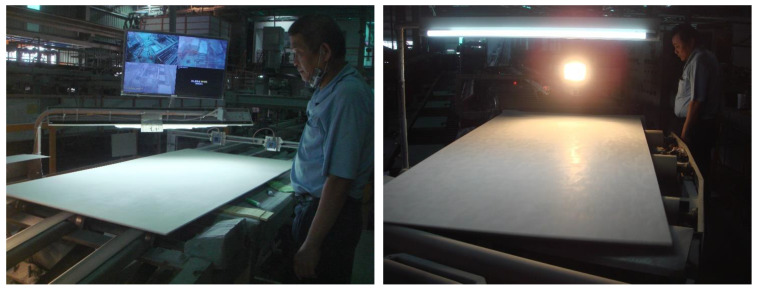
Manual inspection for surface defect detection of calcium silicate boards.

Today, Artificial Intelligence (AI) has become the mainstay keystone of industrial development. Manufacturing plants have replaced their workforces with machines and are moving towards complete automation to achieve the goal of reducing labor costs and improving efficiency. In order to ensure the overall quality of the product after the production, the back-end of the production line usually requires a defect detection process. Currently, many manufacturing industries still use manual inspection for the defect detection. For instance, Figure 1 shows two calcium silicate board (CSB) production lines, each of which requires an operator to manually inspect the surface defects of the CSB. This manual inspection method usually increases labor costs, and it is difficult to maintain 24 h operation. The longer the inspection time, the lower the inspector’s concentration. As a result, the incidences of detection error increase. If some kind of relevant expert systems and detection technologies can be used at this stage, the workforce will focus on system maintenance, error correction, and the subsequent processing. This method not only reduces the burden on inspectors, but also further improves productivity while reducing labor costs.

In this paper, we propose a novel automatic CSB surface defect recognition system based on deep learning technology. The proposed deep Convolutional Neural Network (CNN) model is inspired by the existing SurfNet architecture [1]. On this basis, we designed a new CNN architecture and trained the proposed CNN model for the application of CSB surface defect recognition tasks. Moreover, the proposed CNN model also has a lower computational load to meet the requirement of high-speed recognition processing. Combined with a high-speed image capture platform, an automatic optical surface defect recognition system is implemented to realize the real-time production quality inspection on the production line. The main contributions of this paper are as follows:

1. We propose a new CNN model called SurfNetv2, which improves the existing SurfNet so that it can achieve higher recognition accuracy with higher processing speed.
2. We create a new CSB dataset for training and testing the proposed CNN model. Based on this CSB dataset, the performance of the proposed CNN model is evaluated by comparing it with other state-of-the-art methods.
3. When combined with a high-speed image capture platform, we can realize an automatic optical surface defect recognition system to achieve real-time recognition of CSB surface defects on the production line.

Experimental results show that the proposed SurfNetv2 produces an average recognition accuracy rate of 99.90% and 99.75% in our private CSB dataset and the public NEU dataset, respectively, which clearly outperforms the other five state-of-the-art methods. Moreover, the proposed SurfNetv2 model achieves a real-time computing speed of up to 199.38 fps when processing images with a resolution of 128 × 128 pixels. Resultantly, the proposed CSB surface defect recognition system shows great potential in practical applications.

The remainder of this paper is organized as follows. In Section 2 we conduct a literature review of the latest research and related methods in the field of defect detection. We also discuss how to apply the latest deep learning methods in this field, as well as the changes in classic architectures and CNN networks from the past to the present. In Section 3, we describe the proposed CSB surface defect recognition system, including system hardware and software architecture. In Section 4, we elaborate the proposed CNN model architecture and make its competitive comparison with the current SurfNet. In addition, this section also introduces our network training method. In Section 5, we first introduce the software and computer specifications used in the experiment. Next, we explain the collection process of the private CSB dataset required for this study and how to implement data augmentation on these samples. Finally, we present the experimental results of the private CSB dataset and the public NEU dataset, and discuss the performance of the proposed method based on the experimental results. Section 6 summarizes the contribution of this study. Moreover, we also discuss some possible directions for future research.

## 2. Literature Review

### 2.1. Defect Recognition

Defect recognition has become an important issue in industrial development, and the development of visual inspection methods has a long history. The purpose of this research work is to assist the traditional industrial manufacturers to develop a new automated visual inspection technology and implement applications related to surface defect recognition during the production process. Many image recognition methods based on machine vision and machine learning have been applied to this task. For example, Ma et al. [2] proposed a remote detection system for defect detection on the upper part of glass bottles in production lines. They used eight cameras to capture the grayscale images of the mouth, lip and neck of each bottle. The defect detection process involved two parts. The first part was to detect defects occurring in the mouth and lip of a glass bottle by using the images captured from the top of a bottle. The second part was the detection of the defects occurring in the neck and shoulder of a bottle by processing the images shot on the upper-side of the bottle. Chen et al. [3] proposed a rail surface defect detection system based on the machine vision technology. They proposed an automatic detection algorithm to analyze noise cracks on the surface through image processing and to extract defect areas through an adaptive thresholding process. In addition, a dynamic template was designed to detect continuous crack boundaries based on the morphology of cracks. Fu and Jiang [4] proposed a visual detection and recognition technology for the detection of the surface crack of a steel rail. They firstly used a weighted median filtering algorithm to filter the track detection image, and then used a histogram equalization algorithm to enhance the filtered image. Finally, the crack area was extracted and identified based on a threshold segmenting method. Gao et al. [5] designed an online inspection system based on computer vision to inspect surface defects in the copper strips. First, they calculated the prejudgment coefficient to predict the defects. Next, they designed a fuzzy classifier to identify the type of defect based on multiple features extracted from the surface image. Zhou et al. [6] proposed a defect inspection algorithm for the metal surface defect detection applications. They used an improved Bi-Dimensional Empirical Mode Decomposition (BEMD)-based extracting algorithm to filter out complex textures on the metal surface. Next, they applied a canny edge detection operator to detect the edge information of the defects.

Many studies have attempted to transfer the surface defect images to the frequency domain to analyze and obtain defect features. Wu et al. [7] proposed an automatic recognition technique based on the spectrum image of surface defects of hot rolled strips. They transformed the spatial image into the frequency domain through Fourier transform, and used a genetic algorithm to obtain the feature set of the image. Finally, a Learning Vector Quantization (LVQ) neural network was used to detect the surface defects on hot rolled strips. Yu et al. [8] designed an automatic detection system for the surface defect inspection of small magnetic rings. First, they applied a frequency domain transform on the acquired images. Next, a Butterworth high-pass filter was used to inhibit random textures and background noises of the surface image of the magnetic ring. Then, inverse Fourier transform and reduction operation were sequentially performed on the filtered image. Finally, the surface defect area was segmented using binary-value image segmentation. Choi and Kim [9] proposed a unified approach for the defect detection of surface images. The proposed method consists of a global estimation phase and a local refinement phase. The former roughly estimates defect regions by applying a spectral-based approach in a global manner, while the latter locally refines the estimated regions based on the pixel intensity distributions derived from defect and defect-free regions. However, the proposed method is only applicable to grayscale images and is limited by the environment.

Some studies try to convert surface images to other color spaces in order to improve the detection of defects in color images. Tsai et al. [10] designed a Gabor-filtering approach for automatic defect inspection in colored texture surfaces. The proposed method is based on the energy response of the feature map obtained from the convolution of a Gabor filter with the color image characterized by two chromatic features in the CIE-L*a*b* color space. Chang et al. [11] proposed a clustering-based surface defect detection algorithm for the surface inspection of ice-cream bars. They divided the image of the ice-cream bar into several small regions through image pre-processing. Next, they proposed two region-merging strategies and three constraints to combine these small regions. Finally, the defect regions were identified in the HSV color space. The above methods extend the image defect detection process from gray space to color space, but the computational cost of the image analysis will be greatly increased due to the conversion of the color space.

As to the machine learning based methods, Chu et al. [12] realized a defect feature extraction scheme by building the sampling benchmark scale information for the training dataset and using two gradient-based co-occurrence matrices. Next, they used K-nearest neighbor and R-nearest neighbor algorithms to prune the training dataset to improve the learning of the least squares twin support vector machine classifier for strip surface defect detection.

### 2.2. Deep Learning Method

In recent years, the rise of deep learning methods has quickly become the focus of AI. Several surface defect recognition methods based on deep learning have been proposed in the literature, and we divided these methods into three categories. The first category was to treat the defect detection task as a semantic segmentation task. Tao et al. [13] designed a novel CAScaded Auto-Encoder (CASAE) architecture, which is capable of segmenting and classifying defect regions through a CNN model. Although the network architecture of the CASAE model is relatively large, it can improve the adaptability of the network to external factors. Sison et al. [14] proposed a copper clad lamination surface defect detection system, which applies the smoothing filters to eliminate noise from the surface image while segmenting the defect region from background texture. The authors created a CNN model to learn the local features of surface defects and background texture. After training the CNN model, the defects and background images from the segmentation step can be input into the CNN to perform the classification task. Qiu et al. [15] proposed an efficient deep learning-based pixel-wise surface defect segmentation algorithm, which consists of a lightweight Fully Convolutional Network (FCN) to make a pixel-wise prediction of the defect areas. Then, a guided filter is used to refine the contour of the defect area to reflect the real abnormal region. However, the CNN architecture used in this method was very large and consumed more computing resources. Furthermore, this method is not necessarily applicable to other types of surface defect detection tasks. Chen et al. [16] proposed a CNN-based defect detection scheme to detect surface scratches on plastic housings. This method is based on pixel annotation for label training, and uses a sliding window strategy for block cropping, so that the CNN model can effectively mark the defect location in the input image. Mei et al. [17] proposed an unsupervised-learning-based approach to detect and localize defects with only defect-free samples for model training. They used multiple convolutional denoising auto-encoder networks to reconstruct multiscale residual maps, which can be used as the indicator for direct pixel-wise defect prediction. They also extended this approach to the application of automatic detection of fabric defects [18].

The second category of surface defect recognition methods is based on an object detection network which provides object location and classification information. For instance, Wei and Bi [19] proposed a surface defect detection network based on Faster RCNN [20] to perform multi-scale detection on defects of various sizes and types on the surface of aluminum profiles. He et al. [21] proposed a CNN-based surface defect detection approach, which uses a Multilevel-feature Fusion Network (MFN) to combine multiple hierarchical features into a multilevel feature. Based on this multilevel feature, a Region Proposal Network (RPN) is adopted to generate Regions of Interest (ROI). For each ROI, a defect detector consisting of a classifier and a bounding box regressor produces the final detection results. Yanan et al. [22] proposed a surface defect detection method based on the YOLOv3 algorithm to detect defects on the rail surface. They used the idea of transfer learning to apply the YOLOv3 detection model [23] to the defect detection of rail surface. However, if the object detection method is used to detect surface defects, the system must include a bounding box regression network. Therefore, this method is not suitable for defect recognition tasks that do not require defect position information.

The third category of surface defect recognition is to treat it as an object recognition task. Some novel CNN architectures have been proposed for defect recognition research. Azizah et al. [24] proposed a CNN-based mangosteen detection method. Moreover, they used 4-fold cross validation to validate data accuracy of the proposed CNN model. In [1], Arikan et al. proposed a new CNN architecture called SurfNet, which is much smaller than most existing CNN architectures. The authors used the Generative Adversarial Networks (GAN) technology [25] to augment training data and identify surface defects in a variety of materials. Because SurfNet has a small number of parameters, it can effectively increase the processing speed and realize the ability of real-time recognition on the production line. Cheon et al. [26] proposed an automatic defect detection system, which adopts a single CNN model to extract effective features for wafer surface defect classification. The proposed method can identify new defect classes by comparing the CNN features of the unknown classes with the CNN features of the trained classes. In [27], Kim et al. proposed a CNN-based Siamese neural network model [28] to classify steel surface defects based on few-shot learning, which only requires a few images to train the network model. In [29], Ren et al. proposed a three-stage deep learning algorithm to detect bubbles in engines. In the first stage, an auto-encoder was trained using the normal *X*-ray images. The second stage of training only adjusts the weights of a FCN-based binary classifier using both normal and defect images, and the final stage of training can act as a fine-tuning step to further optimize the whole network. In [30], Zhao et al. proposed a defect detection framework only based on the training of positive samples. They combined GAN and an auto-encoder to establish a reconstruction network, which can repair defect areas in the input image. Finally, through a simple comparison between the input image and the reconstructed image, all defect areas can be accurately classified.

Some research is devoted to improving the capabilities of existing methods for defect classification tasks. Kim et al. proposed a CNN-based classification system, which can extract the chip region and improve the color distribution through a color enhancement process [31]. The proposed method extracts the correct chip area by vertical and horizontal projection, and enhances the brightness value distribution of the chip image by local histogram stretching. Gao et al. [32] proposed a multi-level information fusion-based method for vision-based defect recognition. They introduced a three-level Gaussian pyramid to generate multi-level information of defects, and established three VGG16 [33] networks to learn the information and predict the final recognition result. Lu et al. [34] used pix2pix GAN to generate more defect images to adjust the data distribution for class imbalance. Then, they used the Dense Convolutional Network (DenseNet) [35] as the classifier model to obtain a better result of surface defect classification with manipulated data. Guan et al. [36] proposed a novel recognition algorithm for steel surface defects. They used VGG19 as a pre-training model for the steel surface defect classification task, and established a DeVGG19 model to extract feature images in different layers from the defect weight model. Then, they evaluated a feature image quality and adjusted the parameters and structure of VGG19 to design a new VSD network model.

### 2.3. Convolutional Neural Network

Over the past few years, there have been many innovative designs in the development of CNN models, and important breakthroughs in image recognition applications. Among these new designs, VGG is one of the classic CNN models, which increases the receiving field and model nonlinearity by stacking many 3 × 3 convolution kernels. It is well known that the deeper the network, the higher the accuracy that can be obtained, but the loss of data information will also increase as the network depth increases. Therefore, a deeper CNN network will cause a vanishing gradient problem, thereby increasing the difficulty of the network training process. In order to solve this problem, some studies have tried to add batch normalization [37] or dropout [38] methods to prevent gradient vanishing and model overfitting. On the other hand, the authors in [39] and [40] proposed a new ResNet architecture, which introduces the concept of residual learning to solve the shortcomings of the loss of features on the deep network layer. By making a shortcut between input and output, ResNet can learn the residual feature based on the input feature while preventing the gradient vanishing problem, so that the network has a better performance. The results prove its ability to perform image recognition tasks. Experimental results prove that ResNet enables the network to be constructed at a deeper level, thereby improving the shortcomings of the original deep CNN. The authors in [35] proposed the DenseNet architecture, which connects each layer to every other layer in a feed-forward manner. In other words, each layer receives the output of all previous layers as its additional input and uses concatenating operation to fuse all the received feature maps together. This design enables DenseNet to use all feature maps more efficiently, resulting in a better learning ability and training accuracy. However, because DenseNet has a large amount of feed-forward connections, its network architecture is not suitable for extending to deeper networks.

## 3. System Architecture

**Figure 2:**
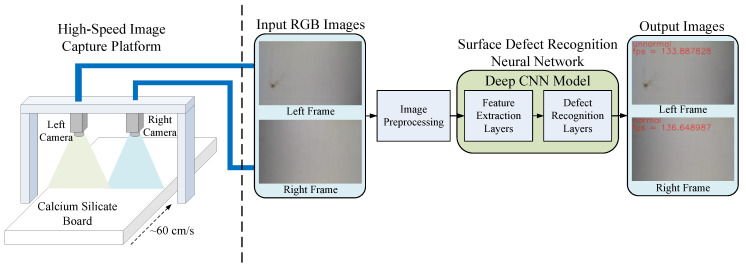
System architecture of the proposed surface defect recognition system.

**Figure 3:**
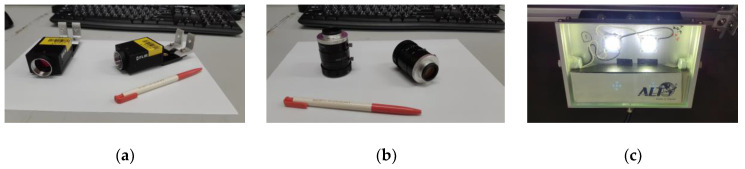
The hardware equipment used in the proposed system: (**a**) two high-speed global-shutter cameras (Grasshopper3, USB3.0, 2.3 million), (**b**) two camera lenses (1”12mm, F1.4, C-mount), and (**c**) four DC LED light sources.

In this section, we introduce the design of the proposed system. Figure 2 shows the system architecture of the proposed surface defect recognition system, which consists of two parts: the hardware part of the high-speed image capture platform and software part of the neural network for the surface defect recognition. In the scenario under consideration, the moving speed of the CSB on the production line is about 60 cm per second, and the operator must complete the defect inspection task within 2 to 3 s. CSBs with defective surfaces are classified as unqualified samples, which can be considered as a real-time defect recognition issue. In order to provide high-quality image capture results, the hardware design of the high-speed image capture platform employs two high-speed global-shutter cameras and four light sources. Suppose that the test field is on a conveyor belt on the production line. We installed the two cameras above the conveyor belt to capture the surface image of the CSB moving under the designed image capture platform. We also installed four light sources near the two cameras so that the surface image of the CSB can be captured quickly and clearly. Figure 3 shows the hardware equipment used in the proposed system, including two high-speed global-shutter cameras (Figure 3a), two camera lenses (Figure 3b), and four DC LED light sources (Figure 3c).

In the software part, the images captured by the two high-speed cameras were input into the computer through the USB3.0 interface, where the image resolution was set to 1920 × 1200 and the frame rate was set to 162 frames per second. When the two captured images were received, we individually performed image pre-processing on the two images, including image resize and pixel normalization. Next, the two normalized images were input into the proposed deep CNN model, which consists of feature extraction layers and defect recognition layers to perform feature extraction and surface defect recognition, respectively. Finally, the proposed system displayed the defect recognition results and the system processing speed on the monitor screen. At the same time, the computer also sent the recognition results to subsequent applications for corresponding processing, such as recycling the CSB with surface defects.

## 4. The Proposed Method

In this section, we introduce the design of our defect recognition model and compare it with the existing SurfNet model. We also explain the training method of the proposed model in this section.

### 4.1. Neural Network Architecture

Based on the concept of the network structure design proposed in [1], we designed the neural network architecture of the proposed CNN model, which can be divided into two parts: feature extraction layers and defect recognition layers. In this section, we introduce the proposed network architecture one by one, and explain the upgrade features of the proposed architecture over the existing SurfNet.

**Figure 4:**
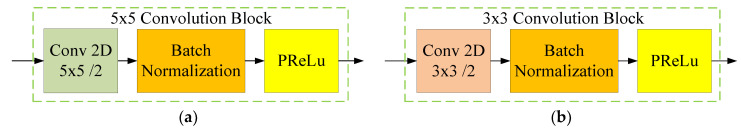
Comparison of the basic convolution block used in (**a**) SurfNet and (**b**) the proposed SurfNetv2.

SurfNet is developed based on the VGG model and residual learning architecture. Figure 4a shows the basic convolution block used in SurfNet for feature extraction. The two-dimensional (2D) convolution uses a fixed 5 × 5 kernel size with padding to gain larger receptive fields and uses down-sampling with a stride step of 2. The output of the 2D convolution is connected to the batch normalization layer, and the final output result is generated via the PReLU [41] activation function defined as follows:

(1) $$\mathrm{PReLU}(x)=\{\begin{array}{cc} x & if\;x>0,\\ ax & if\;x\leq 0, \end{array}$$

where *x* is the input of the nonlinear activation and *a* is a trainable coefficient that controls the slope of the negative part. If *a* is equal to zero, then the PReLU activation function becomes the traditional ReLU [42] function given by:

(2) $$\mathrm{ReLU}(x)=\{\begin{array}{cc} x & if\;x>0,\\ 0 & if\;x\leq 0. \end{array}$$

According to [1], using the PReLU activation function instead of the ReLU function can use its optimization ability to improve the learning accuracy during training. Note that SurfNet does not use any dropout layers together with batch normalization to prevent the problem of model overfitting. Here, we refer to the basic convolution block in SurfNet as a 5 × 5 convolution block to facilitate subsequent comparisons.

Inspired by VGG, we changed the size of the convolution kernel used in the proposed method to 3 × 3 with a stride step of 2 to perform convolution and increase spatial information. We still connected the batch normalization and PReLU activation function behind the 2D convolution without any dropout layer. Figure 4b shows the basic convolution block used in the proposed SurfNetv2 model. Here, we name this module a 3 × 3 Convolution block.

**Figure 5:**
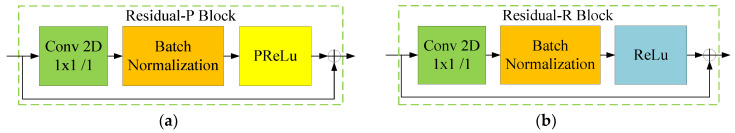
Comparison of the residual block used in (**a**) SurfNet and (**b**) the proposed SurfNetv2.

Figure 5a shows the design of the residual block used in SurfNet. The residual block uses a single 1 × 1 convolution layer without padding, and uses a stride step of 1 for down-sampling. It also uses the batch normalization layer and PReLU activation function after 2D convolution, and realizes the addition of input and output feature maps by a skip connection. Here, we call this block the Residual-P block.

Figure 5b illustrates the proposed residual block, which also uses a 1 × 1 convolution kernel without padding. The stride step of the convolution layer is also 1. The proposed residual block changes the activation function behind the batch normalization layer to the ReLU function to improve the calculation speed and maintain the network learning ability. The rest of this module is the same as the method in [1], and we call it the Residual-R block.

**Figure 6:**
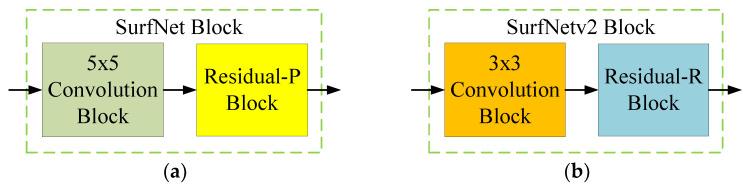
Comparison of (**a**) the original SurfNet block and (**b**) the proposed SurfNetv2 block.

After designing the two types of basic convolutional blocks, we combined them to realize the main convolutional block required in the proposed network architecture. Our design concept is similar to SurfNet, which reduces the dimension of the input feature maps through the convolution block, and then provides additional nonlinearity and residual learning benefits through the residual block. Figure 6a,b show the main convolutional blocks used in the original SurfNet and the proposed SurfNetv2, respectively. We defined the combination of the 5 × 5 convolution block and the Residual-P block as the SurfNet block, and the 3 × 3 Convolution block connecting the Residual-R block was the proposed SurfNetv2 block. Therefore, the difference between the proposed SurfNetv2 block and the original SurfNet block is that we used a 3 × 3 convolutional layer for the convolutional block and a simplified activation function for the residual block.

**Figure 7:**
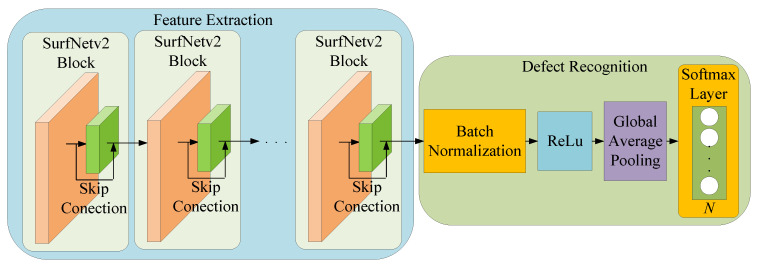
The proposed multi-class surface defect recognition model.

Figure 7 shows the proposed CNN-based multi-class surface defect recognition model, which consists of feature extraction and defect recognition layers. As shown in Figure 7, the proposed feature extraction layers are composed of multiple SurfNetv2 blocks. Because each SurfNetv2 block was implemented by a simple 3 × 3 Convolution block and a Residual-R block, the network architecture of the proposed feature extraction layer is simpler than the general CNN-based backbone models, such as VGG16, ResNet18, etc. According to [34], stacking more network layers can obtain a better recognition ability. Therefore, we built the proposed feature extraction module by stacking multiple SurfNetv2 blocks so that the network can learn more and better features during the down-sampling process.

In many classic CNN models, the output of the final feature extraction layer is usually connected to the flatten layer and at least one Fully Connected (FC) layer to convert 2D feature maps to a 1D feature vector. Finally, the recognition model uses the final FC layer to learn how to classify the input image by the 1D feature vector and the desired output label. However, this approach usually results in a significant increase in the amount of calculations due to a large number of parameters in at least two FC layers, and may lead to the problem of model overfitting. To solve this problem, the authors in [43] proposed a Global Average Pooling (GAP) architecture to effectively reduce the size of multiple feature maps. Assume that the size of *d* feature maps is *h* × *w* × *d*. The GAP layer performs a more extreme type of dimensionality reduction, which simply obtains the average of all h×w feature values to reduce the feature size from *h* × *w* × *d* to 1×1×d, effectively reducing the number of parameters required in the FC layer to prevent the model from overfitting while maintaining the learning ability of the CNN model. Therefore, in the design of the proposed defect recognition layers, we added a GAP layer at the output of the feature extraction network and connected it to the Output Softmax layer, which is an FC layer using the Softmax activation function defined as follows:

(3) $$p(y=j|x)=\frac{\exp(x^{T}w_{j})}{\sum_{i=1}^{N}\exp(x^{T}w_{i})},$$

where ***x*** is the 1D tensor obtained from the GAP layer, **w***_i_* is the weight vector of the *i*-th output of the output FC layer, p(y=j|x) is the conditional probability of the *j*-th category given the tensor vector ***x***, and *N* is the number of categories defined in the training dataset. The Softmax function can compress the range of each element in any *N*-dimensional real vector between 0 and 1, and the sum of all elements is 1. Therefore, it is very suitable for applying to the probability distribution of multiple-class classification. Note that the value of *N* defines the number of neurons in the Output Softmax layer, which can be changed according to the category number of the training dataset. In this study, when using our private CSB defect dataset, the output dimension of the Softmax layer was set to 4, and when using the public NEU dataset [44], the output dimension was set to 6.

**Figure 8:**
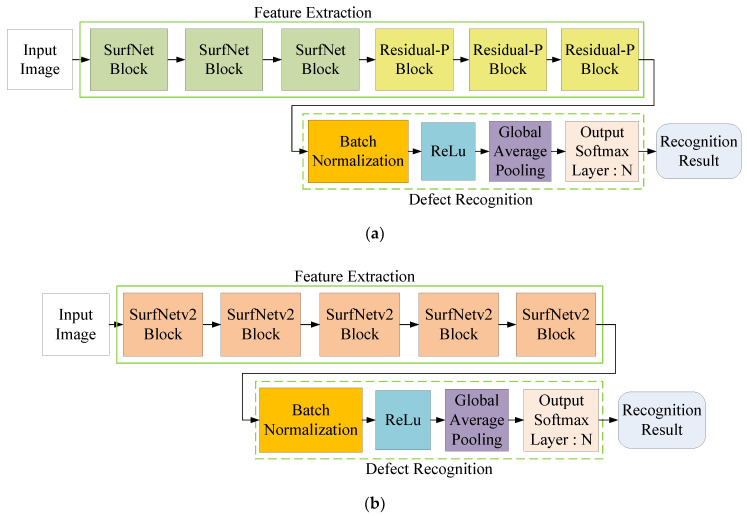
Network architecture of (**a**) the original SurfNet and (**b**) the proposed SurfNetv2 model.

Figure 8 shows a comparison of the network architecture of the original SurfNet (Figure 8a) and the proposed SurfNetv2 model (Figure 8b). As shown in Figure 8a, the feature extraction network used in the SurfNet contains three SurfNet blocks and three Residual-P blocks. The subsequent defect recognition network includes a GAP layer and an Output Softmax layer of dimension *N* to predict the recognition result based on the extracted defect feature maps. However, from experiments, we found that the feature extraction network in SurfNet did not perform well in some surface defect datasets. In order to solve this problem, we replaced the SurfNet block with the proposed SurfNetv2 block and increased the number of blocks to improve the feature extraction capability. In addition, we removed all Residual-P blocks from the feature extraction network. As shown in Figure 8b, the network architecture of the proposed SurfNetv2 model uses a total of five SurfNetv2 blocks in the feature extraction network and maintains the same defect recognition network. Note that the output dimension of the Output Softmax layer can be changed according to the number of categories defined in the training dataset.

We also designed two other SurfNetv2 models with different network architectures to study their recognition performance. Table 1 presents a comparison of the different network architectures of the proposed SurfNetv2 model. As shown in Table 1, all three SurfNetv2 models use the same defect recognition network, which consists of batch normalization, ReLU function, GAP layer, and Output SoftMax layer. On the other hand, all three SurfNetv2 models use different feature extraction blocks. The second model in Table 1 uses the Residual-P block instead of the Residual-R block for the proposed SurfNetv2 block, and we term it the SurfNetv2(RP) model. In contrast, the third model in Table 1 keeps using the Residual-R block, but uses the 5 × 5 Convolution block instead of the 3 × 3 Convolution block for the SurfNetv2 block. We name it the SurfNetv2(5 × 5) model. The last two models might significantly increase the parameters of the CNN model, but they can help us to understand the effect of the PReLU function and the wider receptive field on the defect recognition performance of the proposed SurfNetv2 model.

### 4.2. Model Training

Regarding the parameter setting during the model training phase, we used RMSprop as the optimizer because it can suppress gradient oscillations and deal with complex error surfaces. In this study, the learning rate was set to 1.0 × 10^−^^6^, and the moving average parameter was set to 0.9. We used categorical Cross-Entropy (CE) as the loss function for multi-class classification training. Let $t=[\begin{array}{cccc} t_{1} & t_{2} & \ldots & t_{N} \end{array}]$ denote the 1D tensor of the desired target. The definition of the CE loss associated with the Softmax activation function Equation (3) is given by:

(4) $$CE(t,x)=-\sum_{j=1}^{N}t_{j}\log(p(y=j|x)),$$

where *N* is the category number and *t_j_* is the *j*-th desired output of the 1D target tensor. The batch size was set to 32 for mini-batch training, and the weight decay rate was set to 1.0 × 10^−4^. We followed the parameter initialization method presented in [39] for convolutional layers and batch normalization layers, and the kernel regularizer used L2 regularization. The image resize method used the bilinear interpolation method, and each pixel was divided by 255 for pixel normalization. In the training process, we adjusted the number of epochs according to the convergence of the loss value and accuracy rate. Details of the epoch number setting are explained in the next section.

In order to achieve better training results, K-fold cross-validation is one of the commonly used training methods to avoid model overfitting. This method randomly divides all training data into K sets, one of which is used as the verification data, and all the remaining sets are merged into the training data. We repeated the above processing method until each set had been used as the verification set. In the experiment, we used 10-fold cross-validation to train and verify our method.

## 5. Results and Discussion

In this section, we first introduce the hardware and software information used in this study and explain how we collected CSB defect samples and augmented the data to form our private CSB dataset. Next, we compare the experiment results of the proposed SurfNetv2 model with five state-of-the-art methods, and conduct some discussions based on our observations.

### 5.1. Hardware and Software Specifications

Table 2 shows the computer hardware and software specifications used in the experiment. In the hardware part, we used RTX 2080Ti to train and verify the proposed method in this study. In the software part, we used Ubuntu 18 as the operating system and used Python2 to develop training and testing programs. In addition, we used Keras as the deep learning framework to implement the proposed method and other existing network architectures. The Keras backend is Tensorflow-gpu 1.14.0.

### 5.2. Data Collection and Dataset Creation

**Figure 9:**
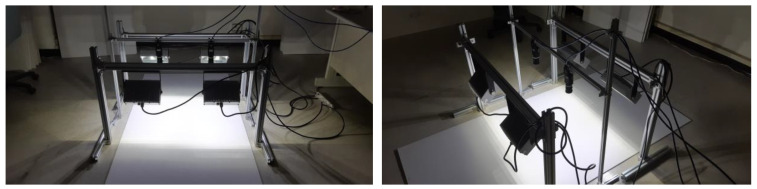
The process of collecting calcium silicate board (CSB) defect samples using the high-speed image capture platform.

We collected CSB defect samples manually using the proposed high-speed image capture platform described in Section 3. We placed the CSB sample under the two cameras, while manually moving the sample to capture defect images of all surfaces on the CSB sample. Figure 9 shows the process of collecting CSB defect samples using the high-speed image capture platform.

**Figure 10:**
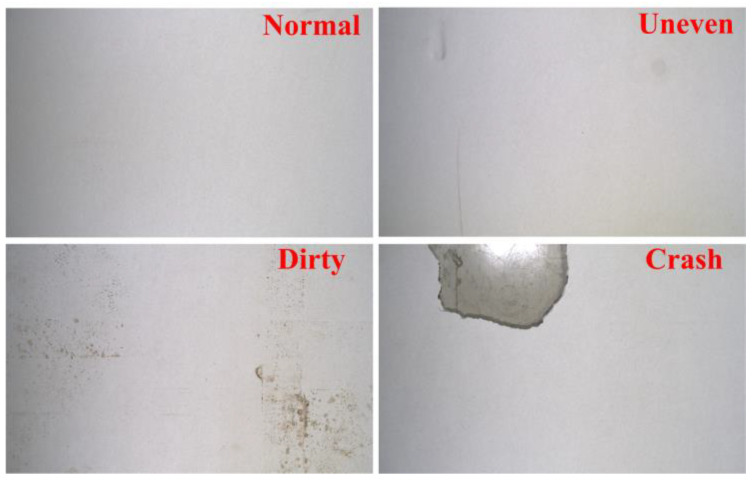
Four categories defined in the private CSB dataset. From top left to bottom right: normal, uneven, dirty, and crash.

**Figure 11:**
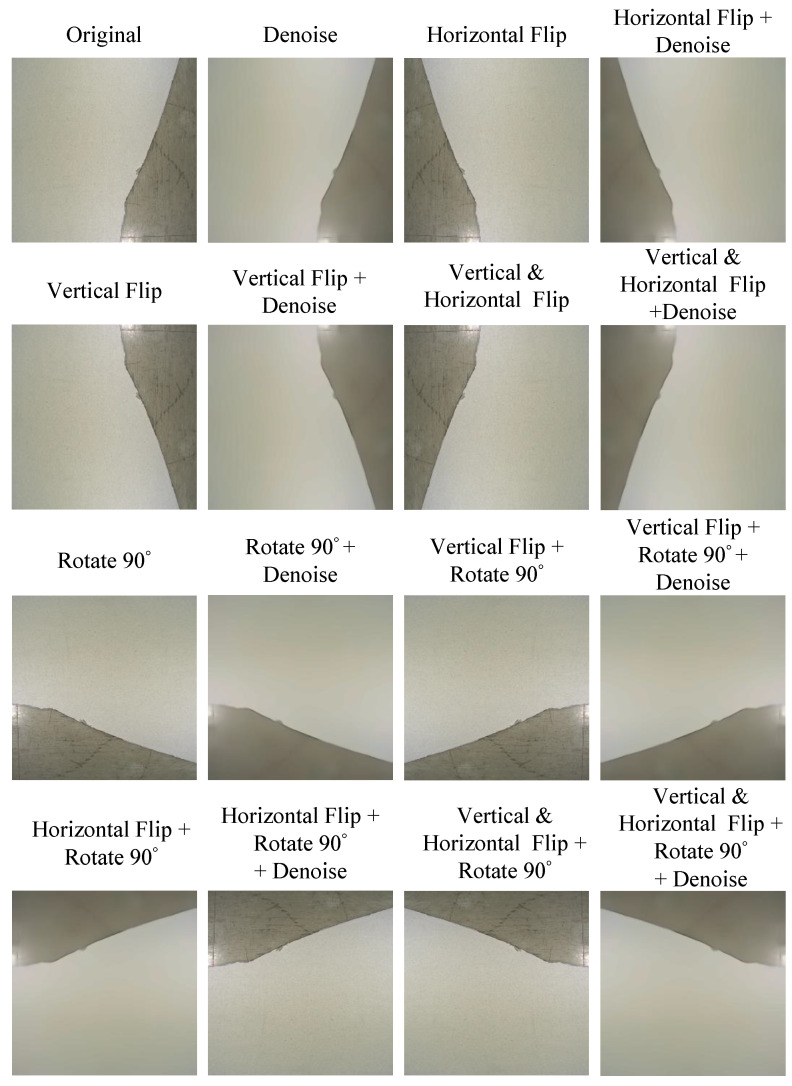
Data augmentation by using fifteen different image processing operations.

After we obtained the preliminary CSB dataset manually, all samples in the dataset were divided into four categories, including normal, uneven, dirty, and crash, as shown in Figure 10. The normal category represented the positive samples in the CSB dataset. Dents and scratches on the CSB surface were classified as the uneven category. Dirt and stains on the surface of CSB were classified as the dirty category. The damage and notches on the surface of CSB were classified as the crash category. Because training a deep CNN model requires a large number of samples, we applied a data augmentation process on the preliminary dataset to increase the number of training samples. The data augmentation method used in this study contained fifteen different image processing operations, which applied different affine transformations, such as rotation or flipping and denoise processing, on the input image to generate multiple images with different views. Figure 11 shows an example of the data augmentation process used in this work. When the number of training samples is large enough, the augmented CSB dataset can be used to train the proposed SurfNetv2 model to learn the optimal model parameters required for the surface defect recognition task.

### 5.3. Training Datasets Used in the Experiment

Two training datasets were used in the experiment, one is the private CSB defect dataset, and the other one is the public NEU dataset. Table 3 shows the sample number of the private CSB dataset. For each category, we manually collected 310 samples and then increased them to 4960 samples through the data augmentation process. Table 4 shows the sample number of the public NEU defect dataset [44], which is a hot-rolled steel strip dataset containing six types of defects. Each category has 300 samples with a size of 200 × 200 pixels. Note that since the number of samples in the CSB and NEU datasets has not reached the level of one million, we chose to use a 10-fold cross-validation method to train and verify the recognition performance of the proposed and compared CNN model.

### 5.4. Performance Evaluation

In the experiment, we compared the proposed SurfNetv2 model with five state-of-the-art methods, namely SurfNet [1], ResNet18 [39], DenseNet [35], VGG16 [33], and MobileNetv2 [45]. As described in Section 4.2, we set the number of epochs based on the convergence of the loss value and accuracy rate of the CNN model during the training process. Table 5 lists the epoch number setting for each model in the CSB and NEU datasets. In the CSB dataset, we selected 200 epochs to train the proposed SurfNetv2 model, because we found that the training results of the proposed model had converged by 200 epochs. Similarly, the epoch number used to train the SurfNet, ResNet18, DenseNet, and VGG16 models was set to 300, 100, 150, and 200 epochs, respectively. In the NEU dataset, we trained the proposed SurfNetv2 model for 400 epochs, and the SurfNet, ResNet18, DenseNet, and VGG16 models for 500, 150, 150, and 300 epochs, respectively. Note that the five comparison methods used the same defect recognition network introduced in Section 4 in order to provide a fair comparison in the experiment.

To evaluate the recognition performance of the CNN model, we used four performance metrics to measure the performance of each CNN model in the training dataset, including Accuracy, Recall, Precision, and F1-measure, which are defined as follows:

(5) $$\mathrm{Accuracy}=\frac{TP+TN}{TP+TN+FP+FN},$$

(6) $$\mathrm{Recall}=\frac{TP}{TP+FN},$$

(7) $$\mathrm{Precision}=\frac{TP}{TP+FP},$$

(8) $$F1-\mathrm{measure}=\frac{2\times \mathrm{Precision}\times \mathrm{Recall}}{\mathrm{Precision}+\mathrm{Recall}},$$

where *TP*, *TN*, *FP*, and *FN* represent the number of True Positive, True Negative, False Positive, and False Negative, respectively. The True Positive and False Positive values, respectively, indicate the number of correct and incorrect positive classifications with respect to the ground truth. In contrast, the True Negative and False Negative values represent the number of correct and incorrect negative classifications relative to the ground truth, respectively. During the K-fold cross-validation training process, we evaluated the training results of each fold based on the four performance metrics defined in Equations (5)–(8). After K trainings, the final performance metrics of the CNN model were obtained by averaging K groups of the four performance metrics.

#### 5.4.1. Private CSB dataset

Table 6 shows the experimental results of the private CSB dataset, in which all the images of the dataset were resized to the size of 128 × 128 and 256 × 256 to observe the effect of different image scales. In Table 6, the bold font indicates the best metric value for each column. By observing Table 6, we have the following findings:

1. All proposed SurfNetv2, SurfNetv2(RP), and SurfNetv2(5 × 5) models performed well on all metrics. Moreover, The SurfNetv2 model with an input size of 128 × 128 obtained the best recognition performance across all metrics, followed by the SurfNetv2(RP) model with input sizes of 128 × 128 and 256 × 256.
2. In addition to the ResNet18 and VGG16 models, the remaining CNN models had better recognition performance when the input size was 128 × 128.
3. The MobileNetv2 model had the least amount of parameters, followed by SurfNet, DenseNet and the proposed SurfNetv2 model. In addition, by observing the parameters of SurfNetv2(RP) and SurfNetv2(5 × 5) models, we found that using 5 × 5 convolution blocks instead of 3 × 3 Convolution blocks greatly increased the network model parameters and reduced the network processing speed. This approach also reduced the recognition performance of the proposed SurfNetv2 model.
4. Although DenseNet requires fewer parameters than the proposed SurfNetv2 model, its processing speed was the slowest one of all comparison methods. The main reason is that DenseNet uses a concatenation operation to perform feature fusion, which makes the computations of each CNN layer greatly increased due to the increase in the number of feature channels, resulting in a slow network processing speed.
5. The VGG16 model with an input size of 128 × 128 had the fastest network processing speed, followed by the proposed SurfNetv2 model, and the existing SurfNet model. However, the VGG16 model had the worst recognition performance in the experiment.
6. By comparing the results of the SurfNetv2 and SurfNetv2(RP) models, the use of the PReLU activation function in the proposed SurfNetv2 block did not have much impact on the recognition results. However, this approach slightly increased the network model parameters and reduced the network processing speed.

#### 5.4.2. Public NEU dataset

Table 7 shows the experimental results of the public NEU dataset. In this experiment, we resized all the images of the dataset to the size of 64 × 64 and 128 × 128 to further study the effect of image scales. Similar to Table 6, the bold font in Table 7 indicates the best metric value for each column. Observing Table 7 has similar findings to the CSB dataset:

1. The proposed SurfNetv2, SurfNetv2(RP), and SurfNetv2(5 × 5) models also performed well on all metrics. Furthermore, the SurfNetv2 model with an input size of 128 × 128 still obtained the best recognition performance across all metrics, followed by the DenseNet and ResNet18 models with the input size of 128 × 128.
2. In addition to the SurfNet and SurfNetv2(5 × 5) models, the remaining CNN models also had better recognition performance when the input size was 128 × 128.
3. The recognition performance of the VGG16 model was also the worst and was greatly affected by the image scale.
4. By observing the results of the SurfNetv2(5 × 5) model, we found that when the input size was 64 × 64, using 5 × 5 Convolution blocks instead of 3 × 3 Convolution blocks could improve the recognition performance of the proposed SurfNetv2 model.
5. By comparing the results of the SurfNet and SurfNetv2(RP) models, when the input size was 64 × 64, using the PReLU activation function in the proposed SurfNetv2 block could provide a similar recognition performance as the original SurfNet model.

Based on the above observations, we can conclude that the proposed SurfNetv2 model with the input size of 128 × 128 provides the best recognition performance in the CSB and NEU datasets, and has a high-speed processing capability and a small amount of network parameters.

### 5.5. Block Number Evaluation

In this section, we study how many SurfNetv2 blocks can provide a better recognition performance. Table 8 records the experimental results of the proposed network using different numbers of SurfNetv2 blocks. In this experiment, we only considered the case where the input size of the proposed SurfNetv2 model was 128 × 128 for both CSB and NEU datasets. Next, the number of SurfNetv2 blocks used in the feature extraction network was increased from 3 to 7 to evaluate the recognition performance of the proposed SurfNetv2 model. It is clear from Table 8 that the use of five SurfNetv2 blocks in the feature extraction network obtained the best recognition performance of the proposed SurfNetv2 model for both CSB and NEU datasets. Therefore, we chose to use five SurfNetv2 blocks in the proposed network.

## 6. Conclusions and Future Work

This paper proposes a real-time surface defect recognition system based on the novel SurfNetv2 model to realize the application of online CSB surface defect recognition. The proposed SurfNetv2 model is developed based on a new SurfNetv2 block, which comprises a 3 × 3 Convolution block and a Residual-R block. In the design of the feature extraction network, we connected multiple SurfNetv2 blocks to improve the feature extraction capability of the network model, thereby improving the recognition performance of the proposed SurfNetv2 model. Experimental results show that the proposed method can perform high-precision classification while maintaining high-speed processing speeds above 190 FPS. Observing the comparison results of the CSB and NEU databases, it can be found that when the input size is 128 × 128, the proposed method has the best recognition performance and is superior to five state-of-the-art methods, including SurfNet, DenseNet, ResNet18, VGG16, and MobileNetv2. Therefore, the recognition performance of the proposed method is verified, and it has great potential for real-time automatic surface defect recognition applications.

In the future, we will try to extend the proposed CNN model to design a new type of defect detection network and apply it to related applications. In addition, how to apply the proposed CNN model to defect detection applications under different light source conditions is a direction worth studying.

## Figures and Tables

**Table 1:** Different network architectures of the proposed SurfNetv2 model.

Network Module	Feature Extraction					Detect Recognition
Block Name	Block1	Block2	Block3	Block4	Block5	Output
SurfNetv2	3 × 3 Convolution Block					BN, ReLU, GAP [43], Output SoftMax
	Residual-R Block					
SurfNetv2(RP)	3 × 3 Convolution Block					
	Residual-P Block					
SurfNetv2(5 × 5)	5 × 5 Convolution Block					
	Residual-R Block					

**Table 2:** Hardware and software specifications.

Part	Item	Content
Hardware	CPU	IntelR Xeon(R)E5-2630 v3
	RAM	32GB
	GPU	RTX 2080Ti
Software	System	Ubuntu 18.04 LTS
	Tool	Python 2.7.17
	Tool	Keras
	Backend	Tensorflow-gpu 1.14.0

**Table 3:** Private CSB defect dataset.

Class	Sample Number	
	Manual Collection	Data Augmentation
Crash	310	4960
Dirty	310	4960
Uneven	310	4960
Normal	310	4960

**Table 4:** Public NEU defect dataset.

Class	Sample Number
Rolled-in Scale (RS)	300
Patches (Pa)	300
Crazing (Cr)	300
Pitted Surface (PS)	300
Inclusion (In)	300
Scratches (Sc)	300

**Table 5:** Epoch number setting in two different datasets for each model.

Model	Epoch Number	
	CSB Dataset	NEU Dataset
SurfNetv2	200	400
SurfNetv2(PP)		
SurfNetv2(5 × 5)		
SurfNet [1]	300	500
ResNet18 [39]	100	150
DenseNet [35]	150	150
VGG16 [33]	200	300
MobileNetv2 [45]	200	300

**Table 6:** Experiment results of the private CSB dataset.

Model	Image Size	Accuracy	Recall	Precision	F1-Measure	FPS	Parameters
SurfNetv2	128 × 128	**99.90%**	**99.89%**	**99.90%**	**99.90%**	199.38	8.2M
	256 × 256	99.83%	99.83%	99.84%	99.84%	157.91	
SurfNetv2(RP)	128 × 128	99.88%	99.88%	99.88%	99.88%	182.68	8.7M
	256 × 256	99.86%	99.86%	99.87%	99.86%	153.15	
SurfNetv2(5 × 5)	128 × 128	99.85%	99.85%	99.85%	99.85%	123.07	19.3M
	256 × 256	99.80%	99.80%	99.80%	99.80%	114.77	
SurfNet	128 × 128	99.68%	99.65%	99.70%	99.68%	198.93	2.4M
	256 × 256	99.33%	99.22%	99.39%	99.31%	154.21	
ResNet18	128 × 128	99.79%	99.79%	99.80%	99.79%	142.36	11.1M
	256 × 256	99.82%	99.81%	99.82%	99.82%	124.21	
DenseNet	128 × 128	99.71%	99.71%	99.71%	99.71%	42.77	7.0M
	224 × 224	99.37%	99.37%	99.38%	99.38%	40.20	
VGG16	128 × 128	83.45%	83.38%	83.53%	83.45%	**230.07**	14.7M
	256 × 256	85.88%	85.77%	85.92%	85.84%	128.05	
MobileNetv2	128 × 128	97.28%	97.22%	97.34%	97.28%	97.42	**2.2M**
	256 × 256	98.37%	98.35%	98.39%	98.37%	89.64	

**Table 7:** Experiment results of the public NEU dataset.

Model	Image Size	Accuracy	Recall	Precision	F1-Measure
SurfNetv2	64 × 64	99.37%	99.37%	99.44%	99.40%
	128 × 128	**99.75%**	**99.75%**	**99.75%**	**99.75%**
SurfNetv2(RP)	64 × 64	99.38%	99.31%	99.38%	99.34%
	128 × 128	99.56%	99.56%	99.56%	99.56%
SurfNetv2(5 × 5)	64 × 64	99.44%	99.44%	99.44%	99.44%
	128 × 128	99.44%	99.38%	99.44%	99.41%
SurfNet	64 × 64	99.37%	99.25%	99.44%	99.34%
	128 × 128	99.31%	99.25%	99.44%	99.34%
ResNet18	64 × 64	99.50%	99.31%	99.55%	99.43%
	128 × 128	99.62%	99.62%	99.69%	99.66%
DenseNet	64 × 64	99.06%	98.94%	99.43%	99.17%
	128 × 128	99.62%	99.62%	**99.75%**	99.69%
VGG16	64 × 64	95.94%	95.19%	96.36%	95.76%
	128 × 128	98.00%	97.81%	98.17%	97.99%
MobileNetv2	64 × 64	94.38%	93.19%	94.84%	93.94%
	128 × 128	96.94%	96.62%	97.24%	96.92%

**Table 8:** Recognition performance of the proposed network using different numbers of SurfNetv2 blocks.

Dataset	Image Size	Block Number	Accuracy	Recall	Precision	F1-Measure
Private CSB	128 × 128	3	98.72%	98.36%	98.98%	98.66%
		4	99.74%	99.70%	99.76%	99.73%
		**5**	**99.90%**	**99.89%**	**99.90%**	**99.90%**
		6	99.82%	99.82%	99.82%	99.82%
		7	99.64%	99.64%	99.64%	99.64%
Public NEU	128 × 128	3	96.94%	96.19%	97.74%	96.93%
		4	99.38%	99.06%	99.43%	99.25%
		**5**	**99.75%**	**99.75%**	**99.75%**	**99.75%**
		6	98.94%	98.88%	98.94%	98.90%
		7	98.44%	98.44%	98.50%	98.47%

## References

[1] Arikan S., Varanasi K., Stricker D. (2019). Surface defect classification in real-time using convolutional neural networks. arXiv.

[2] Ma H.-M., Su G.-D., Wang J.-Y., Ni Z. A glass bottle defect detection system without touching. Proceedings of the International Conference on Machine Learning and Cybernetics.

[3] Chen L., Liang Y., Wang K. Inspection of rail surface defect based on machine vision system. Proceedings of the 2nd International Conference on Information Science and Engineering.

[4] Fu S., Jiang Z. Research on image-based detection and recognition technologies for cracks on rail surface. Proceedings of the 2019 International Conference on Robots & Intelligent System (ICRIS).

[5] Gao F., Li Z., Xiao G., Yuan X., Han Z. An online inspection system of surface defects for copper strip based on computer vision. Proceedings of the 2012 5th International Congress on Image and Signal Processing.

[6] Zhou A., Zheng H., Li M., Shao W. Defect inspection algorithm of metal surface based on machine vision. Proceedings of the 2020 12th International Conference on Measuring Technology and Mechatronics Automation (ICMTMA).

[7] Wu G., Zhang H., Sun X., Xu J., Xu K. A bran-new feature extraction method and its application to surface defect recognition of hot rolled strips. Proceedings of the 2007 IEEE International Conference on Automation and Logistics.

[8] Yu Z., Li X., Yu H., Xie D., Liu A., Lv H. Research on surface defect inspection for small magnetic rings. Proceedings of the 2009 International Conference on Mechatronics and Automation.

[9] Choi J., Kim C. Unsupervised detection of surface defects: A two-step approach. Proceedings of the 2012 19th IEEE International Conference on Image Processing.

[10] Tsai D., Lin C., Huang K. (2005). Defect detection in colored texture surfaces using Gabor filters. Imaging Sci. J..

[11] Chang Q., Zhang Y., Sun Z. Research on surface defect detection algorithm of ice-cream bars based on clustering. Proceedings of the 2019 IEEE 3rd Information Technology, Networking, Electronic and Automation Control Conference (ITNEC).

[12] Chu M., Wang A., Gong R., Sha M. (2014). Strip steel surface defect recognition based on novel feature extraction and enhanced least squares twin support vector machine. J. Int..

[13] Tao X., Zhang D., Ma W., Liu X., Xu D. (2018). Automatic metallic surface defect detection and recognition with convolutional neural networks. Appl. Sci..

[14] Sison H., Konghuayrob P., Kaitwanidvilai S. A convolutional neural network for segmentation of background texture and defect on copper clad lamination surface. Proceedings of the 2018 International Conference on Engineering, Applied Sciences, and Technology (ICEAST).

[15] Qiu L., Wu X., Yu Z. (2019). A high-efficiency fully convolutional networks for pixel-wise surface defect detection. IEEE Access.

[16] Chen Y.-F., Yang F.-S., Su E., Ho C.-C. Automatic defect detection system based on deep convolutional neural networks. Proceedings of the 2019 International Conference on Engineering, Science, and Industrial Applications (ICESI).

[17] Mei S., Yang H., Yin Z. (2018). An unsupervised-learning-based approach for automated defect inspection on textured surfaces. IEEE Trans. Instrum. Meas..

[18] Mei S., Wang Y., Wen G. (2018). Automatic fabric defect detection with a multi-scale convolutional denoising autoencoder network model. Sensors.

[19] Wei R., Bi Y. (2019). Research on recognition technology of aluminum profile surface defects based on deep learning. Materials.

[20] Ren S., He K., Girshick R., Sun J. Faster R-CNN: Towards real-time object detection with region proposal networks. Proceedings of the Advances in Neural Information Processing Systems.

[21] He Y., Song K., Meng Q., Yan Y. (2019). An end-to-end steel surface defect detection approach via fusing multiple hierarchical features. IEEE Trans. Instrum. Meas..

[22] Yanan S., Hui Z., Li L., Hang Z. Rail surface defect detection method based on YOLOv3 deep learning networks. Proceedings of the 2018 Chinese Automation Congress (CAC).

[23] Redmon J., Farhadi A. (April 2018). YOLOv3: An Incremental Improvement.

[24] Azizah L.M., Umayah S.F., Riyadi S., Damarjati C., Utama N.A. Deep learning implementation using convolutional neural network in mangosteen surface defect detection. Proceedings of the 2017 7th IEEE International Conference on Control System, Computing and Engineering (ICCSCE).

[25] Isola P., Zhu J.-Y., Zhou T., Efros A.A. Image-to-image translation with conditional adversarial networks. Proceedings of the IEEE Conference on Computer Vision and Pattern Recognition.

[26] Cheon S., Lee H., Kim C.O., Lee S.H. (2019). Convolutional neural network for wafer surface defect classification and the detection of unknown defect class. IEEE Trans. Semicond. Manuf..

[27] Kim M.S., Park T., Park P. Classification of steel surface defect using convolutional neural network with few images. Proceedings of the 2019 12th Asian Control Conference (ASCC).

[28] Bromley J., Guyon I., LeCun Y. (1993). Signature verification using a “Siamese” time delay neural network. Int. J. Pattern Recognit. Artif. Intell..

[29] Ren J., Ren R., Green M., Huang X. Defect detection from X-Ray images using a three-stage deep learning algorithm. Proceedings of the 2019 IEEE Canadian Conference of Electrical and Computer Engineering (CCECE).

[30] Zhao Z., Li B., Dong R., Zhao P. A surface defect detection method based on positive samples. Proceedings of the Pacific Rim International Conference on Artificial Intelligence.

[31] Kim Y.-G., Lim D.-U., Ryu J.-H., Park T.-H. SMD defect classification by convolution neural network and PCB image transform. In Proceeding of the 2018 IEEE 3rd International Conference on Computing, Communication and Security (ICCCS).

[32] Gao Y., Gao L., Li X., Wang X.V. (2019). A multilevel information fusion-based deep learning method for vision-based defect recognition. IEEE Trans. Instrum. Meas..

[33] Simonyan K., Zisserman A. Very deep convolutional networks for large-scale image recognition. Proceedings of the International Conference on Learning Representations.

[34] Lu Y.-W., Liu K.-L., Hsu C.-Y. Conditional generative adversarial network for defect classification with class imbalance. Proceedings of the 2019 IEEE International Conference on Smart Manufacturing, Industrial & Logistics Engineering (SMILE).

[35] Huang G., Liu Z., van der Maaten L. Densely connected convolutional networks. Proceedings of the IEEE Conference on Computer Vision and Pattern Recognition.

[36] Guan S., Lei M., Lu H. (2020). A steel surface defect recognition algorithm based on improved deep learning network model using feature visualization and quality evaluation. IEEE Access.

[37] Ioffe S., Szegedy C. Batch normalization: Accelerating deep network training by reducing internal covariate shift. Proceedings of the 32nd International Conference on Machine Learning.

[38] Hinton G.E., Srivastava N., Krizhevsky A., Sutskever I., Salakhutdinov R.R. (2012). Improving neural networks by preventing co-adaptation of feature detectors. arXiv.

[39] He K., Zhang X., Ren S., Sun J. Deep residual learning for image recognition. Proceedings of the IEEE Conference on Computer Vision and Pattern Recognition.

[40] He K., Zhang X., Ren S., Sun J. (2016). Identity mappings in deep residual networks. arXiv.

[41] He K., Zhang X., Ren S., Sun J. Delving deep into rectifiers: Surpassing human-level performance on ImageNet classification. Proceedings of the IEEE International Conference on Computer Vision.

[42] Nair V., Hinton G.E. Rectified linear units improve restricted Boltzmann machines. Proceedings of the 27th International Conference on Machine Learning.

[43] Lin M., Chen Q., Yan S. Network in network. Proceedings of the International Conference on Learning Representations.

[44] Song K., Yan Y. (2013). A noise robust method based on completed local binary patterns for hot-rolled steel strip surface defects. Appl. Surf. Sci..

[45] Sandler M., Howard A., Zhu M., Zhmoginov A., Chen L.-C. MobileNetV2: Inverted residuals and linear bottlenecks. Proceedings of the IEEE Conference on Computer Vision and Pattern Recognition.

